# Effectiveness of a peer-to-peer, self-management intervention for young people with depression and anxiety in Denmark: an observational study and a propensity score-matched comparison

**DOI:** 10.3389/fpubh.2024.1377105

**Published:** 2024-10-09

**Authors:** Susan Andersen, Lau Caspar Thygesen, Marie Pil Jensen, Sigurd Lauridsen, Anna Paldam Folker, Maj Britt Dahl Nielsen

**Affiliations:** National Institute of Public Health, University of Southern Denmark, Copenhagen, Denmark

**Keywords:** anxiety, depression, young people, self-management, peer-to-peer, effect

## Abstract

**Objective:**

This study investigated the effectiveness of a 7-weeks peer-to-peer program for young people aged 15 to 25 years with depression or anxiety symptoms in Denmark.

**Methods:**

A total of 483 participants (72% women) participated in the program and the evaluation. The participants completed questionnaires at baseline, postintervention, and at 5-month follow-up to assess changes in depression symptoms (using Beck’s Depression Inventory-II), anxiety symptoms (using Spielbergers State–Trait Anxiety Inventory for Adults) and self-efficacy in controlling or managing the illness (using the personal control subscale from the Illness Perception Questionnaire-Revised). Analyses were done using repeated measures mixed linear regression models. Moreover, a register-based matched comparison group was derived as a comparison to assess changes in being in education and employment at 8-month follow-up.

**Results:**

Within the intervention group, both depression and anxiety scores declined across the 5-month follow-up compared to baseline (*b* = −9.6, 95% CI: −11.2, −8.1 for depression symptoms). The self-efficacy score increased from baseline to post-intervention (*b* = 1.4, 95% CI: 1.0, 1.8) and this level was maintained at follow-up. More than half of the participants were in education at baseline while 24% received social transfer payments. Compared with matched comparison group, a lower proportion of the intervention group remaining in education (71% vs. 80%). Among participants who were employed at baseline, a higher proportion of the intervention group were enrolled in education (27% vs. 19%) and were unemployed (14% vs. 4%) compared to the matched comparison group.

**Conclusion:**

This study supports the effectiveness of a peer-to-peer intervention for depression and anxiety symptoms, and self-efficacy in symptom control. However, mixed effects on education and employment were observed, indicating a negative impact on education among those initially enrolled and a higher proportion of employed participants starting education.

## Introduction

Depression and anxiety are common mental disorders in the general population (1). In children and adolescents (10–19 years), the worldwide prevalence is estimated to 6.5% (95% CI 4.7–9.1) for anxiety disorders and 2.6% (95% CI 1.7–3.9) for depressive disorders (2). In the last decade, prevalence rates have been increasing, and in Denmark, where this study took place, the prevalence of young people receiving treatment for depression and/or anxiety tripled from 2006 to 2016 (3). By the age of 18, 15.01% of children and adolescents in Denmark are diagnosed with a mental disorder (4).

These negative trends in mental health might have been exacerbated by the Covid-19 pandemic. Evidence suggests that, globally, 1 in 4 youth experienced clinically elevated depressive symptoms and 1 in 5 experienced clinically elevated anxiety symptoms during COVID-19. These estimates increased over time, double pre-pandemic estimates. Thus, an influx of mental health care utilization could be expected, and allocation of resources to address child and adolescent mental health concerns are an important public health concern (5).

Mental disorders have short-and long-term consequences. Emotional problems in childhood and youth show continuity with emotional problems later in life and have been associated with impaired functioning in adulthood, including working life (6). Previous research has found a prospective association between emotional problems and poor school attainment, especially for depression (7). Depression is currently the leading cause of disability among adolescents (8), and systematics reviews have shown that adolescent depression is associated with poorer academic performance (9) and reduced odds for completing high school and entering postgraduate education (10). As education is associated with long-term economic growth, trends in adolescent mental health therefore have implications at the societal level.

Mental health care utilization among young people remains low, and less than half of young people with mental health problems receive help (11, 12). Neufeld et al. have shown that young people receiving mental health care (including general practitioner or other specialized mental health caregiver) at age 14 years have a lower likelihood of depression by age 17 years. This finding supports the improvement of access to adolescent mental health services (12). Barriers in access to mental health care include poor mental health literacy (i.e., ability to recognize symptoms), stigma and negative attitudes toward mental health care (13, 14).

The term treatment or mental health care covers a wide range of therapies and interventions. The traditional forms, i.e., cognitive therapy and other therapies, share three characteristics related the mode of delivery (typically in person a one-to-one with individual, couple, or family), the setting (clinic or health care facility) and the therapist (highly trained professional, e.g., master’s or doctorial level) (15). One limitation of this model is the degree to which it can be extended to reach the many individuals in need, especially considering current and future shortages of mental health professionals. Kazdin (15) therefore proposes that we develop new models to close the current treatment gap. These model(s) should be focused on:

1. Reach and scalability, i.e., intervention capacity to reach individuals not usually served and to be applied on a larger scale.
2. Affordability, i.e., lower costs compared to traditional models.
3. Expansion of setting, i.e., bringing interventions to everyday settings.
4. Task shifting to increase the number of interventions providers.
5. Feasibility, and flexibility, i.e., ensuring that interventions can be implemented and adapted to local conditions and needs of diverse groups (15).

How these interventions are best delivered requires more research and innovation, and strategies also require evaluation even if randomized controlled trials are not possible (16).

The aim of the present study is to evaluate a peer-to-peer self-management intervention for young people with anxiety and depression. The intervention named LAT is a community-based intervention delivered by non-mental health specialist in a group format based on principles of empowerment and self-efficacy. LAT has previously demonstrated that it has a positive effect on anxiety and depressive symptoms among adults in a randomized controlled trials (RCT) (17).

The evaluation serves two purposes. First, the primary aim was to assess changes in participants’ depressive and anxiety symptoms and self-efficacy in controlling or managing the illness before and after the intervention. We did not have a control group for this purpose Secondly, we aimed to explore whether the intervention could have a positive effect on educational and labor market attainment. For this purpose, we used a register-based matched comparison group design.

## Materials and methods

### Study designs

We used two study designs. An observational before-after study was conducted from 2017 to 2019 in 39 Danish municipalities to evaluate the questionnaire-based outcomes (depression, anxiety and self-efficacy in controlling or managing the illness). The analyses comprise data from three time-points. The participants completed the first questionnaire (baseline) before the intervention, the second questionnaire was fulfilled in the end of the last session of the course (post-intervention) and a third questionnaire was sent out 5 months after baseline.

A propensity score-matched comparison group design was used to evaluate the register-based outcomes (being in education or employment). The linkage of information from various registries was facilitated by the unique personal identification number assigned to every Danish citizen, which intervention participants were asked to provide in the baseline questionnaire. We employed propensity score matching on register-based variables measured before the intervention to create a comparison group. We use this group to estimate the intervention’s effects on being in education or employment 8 months after baseline.

### Participants and recruitment

The target group for the program is young people aged 15–25 who have symptoms of anxiety and/or depression. The recruitment of participants was undertaken by coordinators designated by Danish municipalities (local governments), using methods such as posters, advertising on social media, and outreach at general practitioners’ offices and schools. Before a participant was granted a place in the course, they had a pre-meeting with a municipal coordinator. At this meeting, the municipal coordinator assessed whether the young people met the following inclusion criteria: self-reported symptoms of anxiety and/or depression, or previous experiences with anxiety and/or depression; willingness to engage in group activities and motivated to work with self-help strategies; ability to comprehend and process information at a level that supports benefiting from the course; voluntary participation; and no signs of aggressive behavior. The screening procedure for including participants has been described in detail elsewhere (18).

### Intervention

The LAT program is a peer-to-peer, group-based program based on the self-management program “New Beginnings” developed by Kate Lorig, Stanford University. This program was provided to the Danish Municipalities by The Danish Committee for Health Education (a non-governmental organization). Young people are highly influenced by peers in values, decision-making and emotional responses. Peer influence on health and well-being is particularly pronounced during this stage of life, suggesting significant potential for peer-to-peer approaches. The program employs a peer-to-peer model in which both instructors and participants have firsthand experience or are currently dealing with symptoms of anxiety and/or depression. Instructors are chosen for their personal experience with these symptoms, although one may have a close relative who does. While the goal was to recruit instructors with direct personal experiences of anxiety and depression, in a few cases this was not possible in practice (18).

The LAT spans 7 weeks, encompassing seven 2.5-h modules, with its content rooted in research literature on self-management and self-efficacy (19). Each module explores 5–7 themes and activities, covering topics such as managing difficult emotions, cognitive techniques, positive thinking, communication, problem-solving, action plans, and collaboration with the healthcare system. Each group involves up to 14 participants and two instructors.

Peer-to-peer instructors, guided by a structured manual, undergo comprehensive training involving two sets of two-day sessions. This training not only educates them on effective instruction but also assesses their ability to serve as positive role models. Throughout the training, instructors receive guidance on constructive interaction with participants. They are certified when completing their training. After 2 years, they receive 2–3 tutorials to be re-certified.

The program is anchored in municipalities (local governments). A local coordinator from the municipal administration was responsible for recruitment. The program was embedded in various municipal settings, such as health centers, labor market administrations, and community psychiatry, adapting to local needs and resources. This approach ensures the program’s accessibility and relevance to diverse communities. Each municipality facilitated between one or two groups.

### Data collection

For the questionnaire-based study digital self-administered questionnaires were used. These questionnaires included questions on socio-demographic, depression, anxiety, and self-efficacy. The first questionnaire was filled out before participation in the course (T1, baseline) during a pre-meeting with the coordinator, who is responsible for both distributing and collecting the questionnaire. The second questionnaire was completed at the end of the last course session (T2, postintervention). Participants who were absent received a link to the questionnaire via email. The third questionnaire was sent out by email 5 months after the start of the course (T3, follow-up).

The data on the participants was transferred to the research servers of Statistics Denmark, who merged them with administrative registers and anonymized the data such that there was still an anonymized identifier of the individual. We had access to detailed register data on (i) educational enrolment and completion with starting and ending dates; (ii) weekly data on types of income transfers received and labor market attainment. In addition to data on education and work status, we had access to a range of variables in several Danish nationwide registers which was utilized to generate the propensity score.

### Outcomes

#### Depressive symptoms

Depressive symptoms were assessed using Beck’s Depression Inventory-II (BDI-II) (20). The BDI-II comprises 21 items, each with four response options on a scale ranging from 0 to 3. The total score ranges from 0 to 63, with higher scores indicating more pronounced symptoms of depression.

#### Anxiety symptoms

To evaluate anxiety symptoms, we used Spielbergers State–Trait Anxiety Inventory for Adults (STAI) (21). STAI comprises 20 items with a four-point Likert scale (from 1 to 4). The total scores range from 20 to 80, higher scores indicating more pronounced anxiety symptoms.

#### Self-efficacy in controlling or managing the illness

Self-efficacy in controlling or managing the illness was assessed using the personal control subscale from the Illness Perception Questionnaire-Revised (IPQ-R) (22). It is composed of six items with a 5-point Likert scale (1 = strongly disagree to 5 = strongly agree), higher total scores representing higher levels of perceived personal control over symptoms.

#### Education or employment

Educational and employment status were obtained from Danish population-based administrative registers. Educational status was identified in the Student Register at Statistics Denmark (23). The Student Register contains individual-level information on all persons enrolled in education. It is a cumulative register which is updated once a year (September 30) based on the educational institutions’ administrative records. The register contents start date for enrollment and end date for graduation or dropout.

Employment status was identified from the register DREAM from the Danish Ministry of Employment which contains data on both employment and use of social welfare transfer payments. DREAM comprises information on the weekly social transfer payments (such as paid long-term sick leave, disability pension, unemployment benefits), and the degree of employment for each month (workhours/month).

We categorized education and employment status into four groups:

(1) In education: registered in the Student Register as enrolled in an education or in the DREAM database as receiving an education grant;
(2) In employment: degree of employment is larger than zero in the DREAM database among persons not enrolled in an education;
(3) Receiving social transfer payments: registered in the DREAM database as receiving social transfer payments (except education grant);
(4) Outside the system: not registered in the Student Register, nor having any employment status, nor receiving social transfer payments in the DREAM database.

### Statistical analysis

Percentages were calculated for demographic variables and education and employment status at baseline, by the study populations for the before-after design and the matched comparison group design, respectively.

For depression and anxiety symptoms and self-efficacy in controlling or managing the illness, repeated measures mixed linear regression models were fitted to the entire longitudinal data. Pairwise comparisons examined differences at postintervention and follow-up relative to baseline (reference timepoint). Values are presented as mean and 95% confidence interval (CI). We performed multiple data imputation to deal with missing data at postintervention and follow-up by imputing missing values in 20 datasets.

#### Matched comparison group design

For the education and employment outcome, we used a propensity score matched comparison group design utilizing data from Danish nationwide registers. The baseline is the date of the pre-interview for the intervention participants (when more than one date was available, the earliest was used), and the matched comparison participants have been assigned the same baseline date.

The comparison group was matched on all known characteristics using propensity scores. We included a broad series of variables in the estimation of propensity including both information on the young person and their parents. Information on the young person included health care use (contact to psychiatric hospital, psychologist, hospital contacts due to anxiety and depression), medication use [sleeping medicine, antipsychotics, antidepressants, sedatives, and attention deficit hyperactivity disorder (ADHD) medications], socioeconomic factors (age, sex, residential situation, urbanization, ethnicity), educational level (highest attained education, grades in compulsory school, interrupted education), income (transfer income, disposable income), and municipal interventions (special education, placed in out of home care). Information on parents included mortality, highest attained education, socioeconomic position, and psychiatric hospital contacts.

We trimmed the dataset by excluding intervention persons with higher propensity score than any reference persons (*n* = 8), and reference persons with lower propensity score than any intervention persons (*n* = 13,607). We conducted a 1:5 match based on the greedy nearest neighbor where for each course participant, the five nearest non-participants were randomly selected. After matching, we compared propensity scores between course participants and the comparison group to assess if there was an overlap. A descriptive analysis has been conducted to assess the extent to which balance has been achieved on all included variables between the intervention and comparison groups.

Subsequently, we performed stratified analyses by baseline educational and employment status (‘in education,’ ‘employed,’ and ‘social transfer payments’). Within each of the three groups, we calculated the proportion who were in education, employed, receiving social transfer payments, or outside the system at the 8-month follow-up. We used chi2 tests to compare the intervention and comparison groups.

Data were analyzed using SAS statistical software, version 9.4 (SAS Institute Inc., Cary, NC, United States).

## Results

A total of 483 young people from 39 municipalities accepted the invitation to participate in the intervention and completed the baseline questionnaire for the evaluation. For the before-after study, 67% (325/483) responded to the postintervention questionnaire and 33% (158/483) responded to the follow-up questionnaire.

For the register-based matched comparison study, 83% (401/483) of the intervention participants were included. The reasons for exclusion were a lack of a full personal identification number (*n* = 48), age ≥ 26 (*n* = 8), participated in more than one course (*n* = 5), and trimming (*n* = 8). Moreover, 13 participants could not be tracked in the administrative registers, possibly because their personal identification number was not typed correctly.

Table 1 shows the characteristics of the student population for the before-after study and the matched comparison group study. Of the study populations, 44–48% were in the age of 18–21 years, and 72% were women. More than half of the study population were in education at baseline and almost one-fourth were unemployed.

**Table 1:** Baseline characteristics of study samples.

	Before-after design	Matched comparison group design	
	Intervention (*n* = 483)	Intervention (*n* = 401)	Reference (*n* = 2005)
	*N* (%)	*N* (%)	*N* (%)
Age group			
<18	117 (24)	123 (31)	600 (30)
18–21	232 (48)	185 (46)	881 (44)
>21	134 (28)	93 (23)	524 (26)
Sex			
Women	346 (72)	289 (72)	1,445 (72)
Men	136 (28)	112 (28)	560 (28)
Highest educational level			
Lower secondary school	–	137 (34)	689 (34)
High school	–	146 (36)	745 (37)
Higher education	–	50 (12)	293 (15)
Unknown	–	68 (17)	278 (14)
Treatment of anxiety or depression			
Psychologist	233 (48)	–	–
Medication	147 (30)	–	–
Psychiatry	138 (29)	–	–
Other	91 (19)	–	–
No form of support	92 (19)	–	–
Education and employment status			
In education	–	210 (52)	1,081 (54)
Employed	–	44 (11)	272 (14)
Receiving social transfer payments	–	99 (24)	472 (24)
Outside the system	–	48 (12)	180 (9)

The variables for the before-after sample were self-reported (questionnaire-based) and the variables for the matched comparison group sample were register-based.

### Anxiety, depression and self-efficacy in controlling or managing the illness over time

There was a decrease on depression symptoms from baseline to postintervention (mean difference = −8.2, 95% CI −9.4, −7.0) and to follow-up (mean difference = −9.6, 95% CI −11.2, −8.1) in the intervention group (Table 2). There was also a decrease on anxiety symptoms from baseline to postintervention (mean difference = −5.8, 95% CI −7.2, −4.4) and to follow-up (mean difference = −6.8, 95% CI −8.6, −5.0) in the intervention group. Self-efficacy in controlling or managing the illness increased from a mean score of 20.3 to a mean score of 21.7 at post-intervention and follow-up assessments (mean difference = 1.4, 95% CI 1.0, 1.8 at post-intervention).

**Table 2:** Change in depression, anxiety and self-efficacy in controlling or managing the illness from baseline to postintervention and follow-up.

	Mean scores at time points			Mean change in score (95% CI)		
	Baseline	Post-intervention	Follow-up	Baseline to post-intervention	Baseline to follow-up	*p-*value^*^
Depression	27.5	19.3	17.9	−8.2 (−9.4, −7.0)	−9.6 (−11.2, −8.1)	<0.0001
Anxiety	52.2	46.4	45.4	−5.8 (−7.2, −4.4)	−6.8 (−8.6, −5.0)	<0.0001
Self-efficacy	20.3	21.7	21.7	1.4 (1.0, 1.8)	1.4 (0.8, 1.9)	<0.0001

* *p-values from repeated measures linear regression models following multiple imputation.*

### Being in education or employment at 8-month follow-up

We found that 71% of the intervention participants who were in education at baseline remained in education at 8-month follow-up, whereas 80% in the comparison group remained in education at 8-month follow-up (*p* < 0.0001) (Table 3).

**Table 3:** Changes in attainment to education and employment, stratified by educational and employment status at baseline.

	In Education at baseline			Employed at baseline			Social transfer payments at baseline		
	Intervention (n = 210)	Reference (n = 1,081)	*p-*value^*^	Intervention (*n* = 44)	Reference (*n* = 272)	*p-*value^*^	Intervention (*n* = 99)	Reference (*n* = 472)	*p-*value^*^
Follow-up (8 months)									
In education	71%	80%	<0.0001	27%	19%	0.02	16%	8%	0.07
Employed	9%	9%		48%	67%		8%	8%	
Social transfer payments	9%	3%		14%	4%		58%	63%	
Outside the system^**^	12%	8%		11%	11%		18%	22%	

^*^Chi^2^ test. ^**^Not registered in education or employment and not receiving financial aid from service institutions.

Among the young people who were employed at baseline, enrollment in education was registered for 27% of the intervention participants compared to 19% among the comparison group, whereas 14% became unemployed in the intervention group compared to 4% in the comparison group (*p* = 0.02).

Among the group of young people who were receiving social transfer payments at baseline, 16% enrolled in education among the intervention group compared to 8% among the comparison group. However, the difference was not statistically significant (*p* = 0.07).

## Discussion

In the current study, there was a decrease in depression and anxiety symptoms and an increase in self-efficacy in controlling or managing the illness in the intervention group from baseline to postintervention, which were maintained at follow-up. Our findings indicate a negative effect on education and employment status among participants being in education at baseline, while a larger proportion of intervention participants in employment at baseline initiated an education, compared with the comparison group.

Our study contributes to knowledge about peer-to-peer interventions targeting young people with anxiety and depression symptoms. Although evidence supports positive effects on depressive symptoms in peer-to-peer interventions targeting (24), very few studies have been conducted among young people (25).

Compared to the previous LAT-intervention for Danish adults over 18 (LAT-18+), we found that the decrease in anxiety and depressive symptoms are similar or higher among the young participants in the present study. Depressive symptoms decreased by 8.2 points at T2 among young participants, compared to 7.1 points among participants in LAT-18+. Likewise, anxiety symptoms decreased by 8.2 points at T2 among young participants, compared to the 5.3 points among LAT18+ participants (17). Although we do not have a control group to establish causal effect of the intervention, the results show improvements in depressive and anxiety symptoms and self-efficacy in managing the illness comparable to those seen in the adults participating in the RCT. Thus, the results suggest that the intervention may also be beneficial for young people.

The results regarding the educational and labor market attainment are somewhat more mixed. Overall, improvements in self-efficacy in controlling or managing the illness and mental health do not seem to translate directly into more favorable educational or work trajectories compared to the comparison group. A recent review of educational- and vocational services targeting young people with psychiatric conditions found that services based on the individual placement and support (IPS) model improved employment outcomes. The authors conclude that adapting the model to include more comprehensive educational support and skills training is important to improve post-secondary educational outcomes (26). Our findings may reflect weaknesses in the design (which we will discuss later) but could also reflect that LAT does not include educational support or vocational skills training (and did not have as goal to improve educational and employment outcomes).

### Strengths and limitations

The strengths of this study include a relative long follow-up period encompassing both immediately postintervention and a longer follow-up assessment and participation from almost half of the municipalities in Denmark. Furthermore, using identical outcome measures as employed in the evaluation of the course among participants aged 18 years or older enhances the comparability and reliability of the findings (17). This study has important limitations to consider. First of all, there was no control group included in the evaluation of changes in depression, anxiety, and self-efficacy; therefore, it is not possible to assess whether these changes would have occurred naturally. It is a common phenomenon that improvement naturally occurs over time. For example, the results from the intervention among adults (LAT-18+), show improvements in the control group as well, even though they did not participate in the course (17). In the evaluation of education and employment status, a strength is the construction of a comparison group that resembles intervention participants on a range of many selected characteristics. This is based on information from various registries, including data on sociodemographic and health, encompassing aspects such as contact with psychiatry, counseling, and the use of specific medications. However, unlike a RCT, the propensity score method ensures comparability only on the variables included in the model, leaving room for potential unobserved differences. A significant challenge is the inability to directly match on anxiety and depressive symptoms since this information is not available in the registries. As not all individuals with symptoms on anxiety or depression seek treatment, we cannot rule out the possibility that the two groups differ in terms of symptom burden. This implies that the observed effects may also reflect differences in the mental health of the intervention and comparison groups at baseline; if there was a higher proportion in the comparison group with lower symptom burden compared to the intervention group, it may obscure the intervention effect, potentially leading to an underestimated impact of the course.

### Implications

The LAT is a tertiary prevention, as it is not designed as an alternative to cognitive behavioral therapy (CBT) or medical treatment. The main aim of the intervention is to help participants manage their symptoms in daily life and reduce anxiety and depressive symptoms. It represents a type of novel intervention delivered by non-health specialists in a community setting and thus has the potential to meet Kazdin’s criteria for effective and scalable mental health promotion interventions: reach and scalability, affordability, everyday settings, task shifting, feasibility, and flexibility (15).

In a comprehensive process evaluation, we found that LAT implementation on a larger scale was feasible, and that the intervention was acceptable for a diverse group of participants (18). Furthermore, we also found that the intervention was likely to be successful in reaching young people who are not receiving more standard types of treatment in the health care system, as 19% of participants did not receive any help to manage their anxiety and depression symptoms at baseline. Among those who did receive mental health care, the most common treatments included therapy delivered by a psychologist (48%), medical treatment (30%), and psychiatric treatment (29%). Thus, the intervention may reach young people who are hesitant to engage in traditional therapy and/or treatment. The participants also highlighted the group format and the peer-to-peer concept, i.e., that the instructors who delivered the intervention themselves had experiences with anxiety and depression symptoms and therefore were better able to understand their situation (18).

However, recruitment and attrition of instructors were found to be challenging. To overcome this challenge some coordinators had to mix instructors with experienced professionals who also had personal experiences with anxiety and depression symptoms and therefore also served as role models. It was also an important finding that peer instructors needed adequate support to ensure that they did not get overwhelmed during the intervention (18).

More generally, peer support does represent an innovative approach to promote the mental health of people with mental health issues. It has found application across diverse contexts, including addiction, substance abuse, and mental health services catering to distinct demographic subgroups such as families, youth, and individuals with disabilities (27, 28). Existing literature indicates that the positive impact of peer support is not confined solely to recipients but also extends to the well-being of the peer support workers themselves. Nevertheless, this promising approach comes with various challenges, encompassing issues like inadequate compensation, social stigma, ambiguous role delineation, feelings of alienation, struggles with skill deficiencies, limited training opportunities, emotional strain associated with aiding others, and the simultaneous task of maintaining personal physical and mental health (29). Unsurprisingly, these hurdles often contribute to elevated attrition rates among peer support workers in mental health settings, as noted in previous studies (29).

The findings of this study did support the effectiveness of LAT as a community-based program tailored to assist young individuals coping with anxiety and/or depression symptoms. Nevertheless, the effects on education and employment yielded mixed results, suggesting a negative influence on the education and employment status of participants who were in education at baseline. Conversely, some positive effects were observed among those who were not enrolled in education at the baseline assessment. Future intervention research should explore the adaptability and implementation of the program within different cultural and organizational settings. The straightforward nature of the intervention suggests its potential applicability and transferability to diverse settings.

## Data Availability

The datasets presented in this article are not readily available because the register-based datasets generated during the current study are not publicly available due to data being stored by Statistics Denmark. Interested readers or researchers must request Statistics Denmark (www.dst.dk) and contact the corresponding author of this study. The questionnaire-based data used for the current study is available from the corresponding author upon reasonable request and with permission from the University of Southern Denmark. Requests to access the datasets should be directed to suan@sdu.dk.

## References

[1] Wittchen HU Jacobi F Rehm J Gustavsson A Svensson M Jönsson B. The size and burden of mental disorders and other disorders of the brain in Europe 2010. Eur Neuropsychopharmacol. (2011) 21:655–79. doi: 10.1016/j.euroneuro.2011.07.018, PMID: 21896369

[2] Polanczyk GV Salum GA Sugaya LS Caye A Rohde LA. Annual research review: a meta-analysis of the worldwide prevalence of mental disorders in children and adolescents. J Child Psychol Psychiatry. (2015) 56:345–65. doi: 10.1111/jcpp.12381 25649325

[3] Danish Health Authority. Prævalens, incidens og aktivitet i sundhedsvæsenet – for børn og unge med angst eller depression, ADHD og spiseforstyrrelser. Copenhagen: Danish Health Authority (2017).

[4] Dalsgaard S Thorsteinsson E Trabjerg BB Schullehner J Plana-Ripoll O Brikell I. Incidence rates and cumulative incidences of the full Spectrum of diagnosed mental disorders in childhood and adolescence. JAMA Psychiatry. (2020) 77:155–64. doi: 10.1001/jamapsychiatry.2019.3523, PMID: 31746968 PMC6902162

[5] Racine N McArthur BA Cooke JE Eirich R Zhu J Madigan S. Global prevalence of depressive and anxiety symptoms in children and adolescents during COVID-19: a meta-analysis. JAMA Pediatr. (2021) 175:1142–50. doi: 10.1001/jamapediatrics.2021.2482, PMID: 34369987 PMC8353576

[6] Rutter M Kim-Cohen J Maughan B. Continuities and discontinuities in psychopathology between childhood and adult life. J Child Psychol Psychiatry. (2006) 47:276–95. doi: 10.1111/j.1469-7610.2006.01614.x, PMID: 16492260

[7] Riglin L Petrides KV Frederickson N Rice F. The relationship between emotional problems and subsequent school attainment: a meta-analysis. J Adolesc. (2014) 37:335–46. doi: 10.1016/j.adolescence.2014.02.010 24793380

[8] WHO. Health for the world’s adolescents: a second chance in the second decade. Geneva: World Health Organization (2014).

[9] Wickersham A Sugg HVR Epstein S Stewart R Ford T Downs J. Systematic review and Meta-analysis: the association between child and adolescent depression and later educational attainment. J Am Acad Child Adolesc Psychiatry. (2021) 60:105–18. doi: 10.1016/j.jaac.2020.10.008, PMID: 33130250 PMC7779367

[10] Clayborne ZM Varin M Colman I. Systematic review and Meta-analysis: adolescent depression and long-term psychosocial outcomes. J Am Acad Child Adolesc Psychiatry. (2019) 58:72–9. doi: 10.1016/j.jaac.2018.07.896, PMID: 30577941

[11] Werlen L Puhan MA Landolt MA Mohler-Kuo M. Mind the treatment gap: the prevalence of common mental disorder symptoms, risky substance use and service utilization among young Swiss adults. BMC Public Health. (2020) 20:1470. doi: 10.1186/s12889-020-09577-6, PMID: 32993605 PMC7526325

[12] Neufeld SAS Dunn VJ Jones PB Croudace TJ Goodyer IM. Reduction in adolescent depression after contact with mental health services: a longitudinal cohort study in the UK. Lancet Psychiatry. (2017) 4:120–7. doi: 10.1016/S2215-0366(17)30002-0, PMID: 28087201 PMC5285445

[13] Gulliver A Griffiths KM Christensen H. Perceived barriers and facilitators to mental health help-seeking in young people: a systematic review. BMC Psychiatry. (2010) 10:113. doi: 10.1186/1471-244X-10-113, PMID: 21192795 PMC3022639

[14] Aguirre Velasco A Cruz ISS Billings J Jimenez M Rowe S. What are the barriers, facilitators and interventions targeting help-seeking behaviours for common mental health problems in adolescents? A systematic review. BMC Psychiatry. (2020) 20:293. doi: 10.1186/s12888-020-02659-0, PMID: 32527236 PMC7291482

[15] Kazdin AE. Addressing the treatment gap: a key challenge for extending evidence-based psychosocial interventions. Behav Res Ther. (2017) 88:7–18. doi: 10.1016/j.brat.2016.06.004, PMID: 28110678

[16] Thapar A Eyre O Patel V Brent D. Depression in young people. Lancet. (2022) 400:617–31. doi: 10.1016/S0140-6736(22)01012-1 35940184

[17] Christensen S Mehlsen MY. Evaluering af satspuljeprojektet: Lær at tackle angst og depression:-En randomiseret kontrolleret undersøgelse. Copenhagen: Sundhedsstyrelsen (2016).

[18] Lauridsen S Nielsen MBD Kusier AO Cloos CØ Jensen MP Andersen S. Implementing a peer-to-peer, self-management intervention for young people with depression and anxiety in Denmark. BMC Psychol. (2022) 10:70. doi: 10.1186/s40359-022-00777-w, PMID: 35296363 PMC8925241

[19] Lorig KR Holman H. Self-management education: history, definition, outcomes, and mechanisms. Ann Behav Med. (2003) 26:1–7. doi: 10.1207/S15324796ABM2601_01, PMID: 12867348

[20] Beck AT Steer RA Brown GK. BDI-II, Beck depression inventory: manual. 2nd ed. San Antonio, Tex., Boston: Psychological Corp. (1996).

[21] Spielberger C Gorsuch R Lushene R Vagg PR Jacobs G. Manual for the State-Trait Anxiety Inventory. Palo Alto, CA: Consulting Psychologists Press (1983).

[22] Moss-Morris R Weinman J Petrie K Horne R Cameron L Buick D. The revised illness perception questionnaire (IPQ-R). Psychol Health. (2002) 17:1–16. doi: 10.1080/08870440290001494

[23] Jensen VM Rasmussen AW. Danish education registers. Scand J Public Health. (2011) 39:91–4. doi: 10.1177/1403494810394715 21775362

[24] Pfeiffer PN Heisler M Piette JD Rogers MAM Valenstein M. Efficacy of peer support interventions for depression: a meta-analysis. Gen Hosp Psychiatry. (2011) 33:29–36. doi: 10.1016/j.genhosppsych.2010.10.002, PMID: 21353125 PMC3052992

[25] Simmons MB Cartner S MacDonald R Whitson S Bailey A Brown E. The effectiveness of peer support from a person with lived experience of mental health challenges for young people with anxiety and depression: a systematic review. BMC Psychiatry. (2023) 23:194. doi: 10.1186/s12888-023-04578-2, PMID: 36964523 PMC10038377

[26] Thompson JL Holloway K Karyczak S Serody MR Lane IA Ellison ML. Evaluating educational and employment services for young people with psychiatric conditions: a systematic review. Psychiatr Serv. (2022) 73:787–800. doi: 10.1176/appi.ps.202000033, PMID: 34875848

[27] Leggatt M Woodhead G. Family peer support work in an early intervention youth mental health service. Early Interv Psychiatry. (2016) 10:446–51. doi: 10.1111/eip.12257, PMID: 26213221

[28] Walker G Bryant W. Peer support in adult mental health services: a metasynthesis of qualitative findings. Psychiatr Rehabil J. (2013) 36:28–34. doi: 10.1037/h0094744, PMID: 23477647

[29] Shalaby RAH Agyapong VIO. Peer support in mental health: literature review. JMIR Ment Health. (2020) 7:e15572. doi: 10.2196/15572, PMID: 32357127 PMC7312261

